# Präklinische Polytraumaversorgung: Beständige Herausforderung im präklinischen Rettungswesen

**DOI:** 10.1007/s00113-023-01383-0

**Published:** 2023-11-09

**Authors:** Daniel Popp, Markus Zimmermann, Maximilian Kerschbaum, Magdalena Matzke, Katrin Judemann, Volker Alt

**Affiliations:** 1 Uniklinik Regensburg, Klinik und Poliklinik für Unfallchirurgie, Franz-Josef-Strauß Allee 11, 93053 Regensburg, Deutschland; 2 Interdisziplinäre Notaufnahme, Universitätsklinik Regensburg, Regensburg, Deutschland; 3 Klinik und Poliklinik für Unfallchirurgie, Universitätsklinik Regensburg, Regensburg, Deutschland; 4 Klinik für Anästhesiologie, Universitätsklinik Regensburg, Regensburg, Deutschland

**Keywords:** Lebenserhaltende Versorgung, Notfallmedizin, Reanimation, Schock, Blutung, Life support care, Emergency medicine, Resuscitation, Shock, Hemorrhage

## Abstract

Tscherne definierte erstmals 1966 das „Polytrauma“ als „mehrere gleichzeitig erlittene Verletzungen verschiedener Körperregionen, wobei mindestens eine Verletzung oder die Kombination dieser Verletzungen lebensbedrohlich ist“. Diese Definition stellt das wesentliche pathophysiologische Paradigma des Polytraumas, die durch die Verletzung mehrerer Organsysteme resultierende Lebensgefahr, heraus. Die Behandlung polytraumatisierter Patienten beginnt am Unfallort. Dort können durch zielgerichtete Maßnahmen des Rettungsteams bereits wichtige lebensrettende Ersteingriffe durchgeführt und das Überleben der Patienten verbessert werden. Weltweiten Standard stellen die Konzepte Advanced Trauma Life Support und Pre Hospital Trauma Life Support (ATLS, PHTLS) dar. Da die präklinische Versorgung des Schwerstverletzten keine Routine bedeutet, sind Konzept und Notfallinterventionen regelmäßig zu trainieren. Nur so ist es möglich, in dieser zeitkritischen Situation effektiv und sicher behandeln zu können.

## Abstract (en)

Tscherne was the first to define the term polytrauma in 1966 as “multiple injuries to different regions of the body sustained simultaneously, with at least one injury or the combination of these injuries being life-threatening”. This definition highlights the essential pathophysiological paradigm of polytrauma, with the life-threating characteristics resulting from injuries to multiple organ systems. The treatment of polytrauma patients begins at the scene of the accident. Important life-saving initial interventions can already be carried out on site through targeted measures and expertise of the emergency medical service team, thus improving patient survival. The advanced trauma life support/prehospital trauma life support (ATLS/PHTLS) concept is the worldwide gold standard. As prehospital treatment of severely injured patients is not routine for most emergency teams, concepts and emergency interventions must be regularly trained. This is the prerequisite for safe and effective emergency treatment in this time-critical situation.

## Lernziele

Nach der Lektüre dieses Beitrags

- verstehen Sie die Inhalte des Advanced Trauma Life Support.
- können Sie multipel auftretende Verletzungen zeitkritisch priorisieren.
- sind Sie mit Möglichkeiten zur präklinischen Blutungskontrolle vertraut.
- verstehen Sie die Besonderheiten der traumabedingten Reanimation.
- kennen Sie Kriterien zur Auswahl einer Zielklinik für einen polytraumatisierten Patienten.

## Einleitung

Die präklinische Erstversorgung eines polytraumatisierten Patienten stellt eine besondere Herausforderung für das Rettungsteam dar. Um die Vielzahl der Verletzungen adäquat behandeln zu können, müssen klare Vorgaben in der **Behandlungsreihenfolge**

Behandlungsreihenfolge

beachtet werden. Neben einem fundierten theoretischen Fachwissen ist ein zügiges, **zielgerichtetes Handeln**

zielgerichtetes Handeln

Voraussetzung für ein möglichst gutes Patienten-Outcome. Ziele der initialen präklinischen Versorgung sind das schnelle Erkennen und die prioritätenorientierte Behandlung akut lebensbedrohlicher Verletzungen.

### Fallbeispiel

Als ersteintreffender Notarzt müssen Sie einen Patienten versorgen, der aus ca. 5–6 m Höhe gestürzt ist. In der ersten klinischen Untersuchung erweist sich der Patient als kardiopulmonal instabil (Blutdruck 75/40 mm Hg, Herzfrequenz [HF] 128 Schläge/min). Bei der Inspektion der Pupillen zeigen sich rechtsseitig eine Lichtstarre und Weitstellung. Der Wert auf der Glasgow Coma Scale (GCS) beträgt 3 Punkte. Das Becken des Patienten imponiert instabil mit einem gespannten Abdomen. Des Weiteren sind die beiden Femora offen frakturiert. Nach der Etablierung eines gesicherten Atemwegs durch eine endotracheale Intubation erfolgt aufgrund eines fehlenden Atemgeräusches und einer pulsoxymetrisch gemessenen Sauerstoffsättigung (S_p_O_2_) von ca. 70 % die Anlage einer Thoraxdrainage. Im Folgenden ist eine deutliche Besserung der Oxygenierung festzustellen. Nach der Anlage von 2 großlumigen venösen Zugängen kann eine Volumensubstitution gestartet werden. Es wird außerdem 1 g Tranexamsäure zur Blutungskontrolle verabreicht. Die klinische Untersuchung ergibt ein instabiles Becken, woraufhin ein Beckengurt angelegt wird. Die Wunden über den offenen Femurfrakturen können mithilfe eines Kompressionsverbands versorgt und der Blutverlust darüber gestoppt werden. Es erfolgen die grob achsengerechten Repositionen der Femora und Immobilisierung des Patienten auf einer Vakuummatratze. Im Rahmen der Inspektion der Patientenrückseite können keine weiteren Verletzungen erkannt werden. Aufgrund des vorliegenden Verletzungsmusters wird eine Schockraumanmeldung durchgeführt [1]. Der Patient wird im Folgenden für den Transport vorbereitet und kann zur Diagnostik, einschließlich schnittbildgebender Untersuchung, luftgebunden in das nächstgelegene überregionale Traumazentrum verbracht werden. Bei Ankunft ist der Patient unter forcierter Volumengabe kardiopulmonal stabil.

## Grundlagen

Ein Polytrauma ist definiert als „mehrere gleichzeitig erlittene Verletzungen verschiedener Körperregionen, wobei mindestens eine Verletzung oder die Kombination dieser Verletzungen lebensbedrohlich ist“ [2]. Der polytraumatisierte Patient und seine Behandlung unterscheiden sich in einigen Faktoren grundlegend vom leicht- oder einzelverletzten Patienten. Der Behandler trifft auf **multiple Verletzungen**

multiple Verletzungen

(z. B. Schädel-Hirn-Trauma [SHT], Thoraxtrauma, Abdominaltrauma, Extremitätenverletzungen) und muss diese am Unfallort zunächst erfassen, einordnen und priorisieren. Grundsätzlich gilt der Leitsatz: „Treat first what kills first“ [3].

Simultan auftretende pathophysiologische Reaktionen (z. B. Hypoxie, Koagulopathie, Acidose, Hypothermie und Schock) müssen berücksichtigt und sollten unter ständiger Reevaluation schnell und effektiv erkannt und behandelt werden. Von besonderer Bedeutung ist die schnelle und zielgerichtete Behandlung eines blutenden schwer verletzten Patienten. Diese komplexe Schockbehandlung wird unter dem Begriff „Damage Control Resuscitation (DCR)“ zusammengefasst [3]. Die gesamte Behandlung unterliegt einem gewissen Zeitdruck, die von Cowley und Dunham treffend als „golden hour in shock“ bezeichnet wurde [4]. Die präklinische Versorgungszeit sollte so kurz wie möglich sein, überlebenssichernde Maßnahmen müssen jedoch durchgeführt werden. Ein **interdisziplinäres Team**

interdisziplinäres Team

ist unabdingbar, um dieser Herausforderung mit hoher Qualität gerecht zu werden [5].

Die Anwendung von Behandlungskonzepten im **Polytraumamanagement**

Polytraumamanagement

konnte in den vergangenen Jahrzenten die Versorgungsqualität und das Outcome schwer verletzter Patienten deutlich verbessern. Insbesondere die flächendeckende Anwendung und Vermittlung der Ausbildungskonzepte „Advanced Trauma Life Support“ (ATLS®) und „Pre Hospital Trauma Life Support“ (PHTLS) als strukturierte, priorisierende Handlungsempfehlungen im Polytraumamanagement stellten einen Meilenstein dar [6]. Zudem führten organisatorische Weiterentwicklungen, wie beispielsweise das *Weißbuch Schwerverletztenversorgung* der Deutschen Gesellschaft für Unfallchirurgie (DGU) [7] oder die heute etablierten Traumanetzwerke deutscher Kliniken zu einer deutlichen Qualitätssteigerung bei der Versorgung polytraumatisierter Patienten (www.traumanetzwerk-dgu.de, [8]). Im Dezember 2022 wurde ein Update der interdisziplinär entwickelten S3-Leitlinie „Polytrauma/Schwerverletzten-Behandlung“ veröffentlicht, auf das sich der vorliegende Beitrag bezieht [1].

## Advanced Trauma Life Support

**Figure 1:**
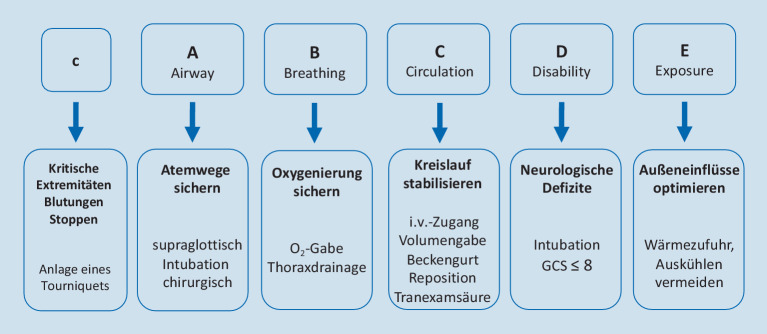

Das ATLS-Konzept (Abb. 1) stellt weltweit den Goldstandard in der prä- und innerklinischen Versorgung polytraumatisierter Patienten dar. Die Behandlungsreihenfolge gemäß ATLS ermöglicht es, auch bei einer Vielzahl lebensbedrohlicher Verletzungen, eine Behandlung der zeitkritischsten Verletzungen zu priorisieren. In den letzten Jahren hat sich das klassische ABCDE-Schema zum **cABCDE-Schema**

cABCDE-Schema

weiterentwickelt.

### c – Critical bleeding

Die Erweiterung um das „c“ beschreibt die Akutversorgung bei katastrophalen Blutungen bzw. drohender Exsanguination, die mithilfe rasch anzuwendender Maßnahmen/Devices (manuelle Kompression, Druckverband ggf. mit Hämostyptikum, Tourniquet) unter Kontrolle gebracht werden sollen [9, 10, 11]. Ein **Tourniquet**

Tourniquet

soll dann angewendet werden, wenn eine vital bedrohliche Blutung mit anderen Maßnahmen nicht zeitgerecht gestoppt werden kann.

#### Cave

- Ziel des „critical bleeding“-Managements ist eine rasche Blutungskontrolle.
- Der Zeitverlust vor einer ggf. notwendigen Atemwegssicherung muss minimal gehalten werden.

### A – Airway

Das **Atemwegsmanagement**

Atemwegsmanagement

hat in der präklinischen Polytraumaversorgung eine hohe Priorität. Es hat die Sicherstellung von **Oxygenierung**

Oxygenierung

und **Ventilation**

Ventilation

zum Ziel. Erfahrungsgrad und Routinetraining des Anwenders, Umstände an der Einsatzstelle (z. B. Einklemmung, Rettungszeit), Transportart (bodengebunden vs. luftgestützt), Transportzeit sowie u. a. Begleitverletzungen im Bereich der Atemwege sind wichtige Aspekte in der Entscheidungsfindung für oder gegen eine **Notfallnarkose**

Notfallnarkose

und **endotracheale Intubation**

endotracheale Intubation

am Einsatzort. Im Fall einer Hypoxie (S_p_O_2_ < 90 %) trotz Sauerstoffgabe, schwerem SHT (GCS < 9 Punkte) oder einer respiratorischen Insuffizienz (Atemfrequenz [AF] < 6 Atemzüge/min oder > 29 Atemzüge/min) sollten eine Notfallnarkose, eine endotracheale Intubation und eine Beatmung durchgeführt werden. Die Bedeutung der **Videolaryngoskopie**

Videolaryngoskopie

wurde in der aktuellen Leitlinie erneut konkretisiert; diese sollte primär eingesetzt werden. Eine **Kapnometrie/-graphie**

Kapnometrie/-graphie

soll zur Tubuslagekontrolle und danach zur Dislokations- und zur Beatmungskontrolle (Normoventilation) angewendet werden [1, 12, 13, 14]. Die **Immobilisation**

Immobilisation

der HWS durch eine Zervikalstütze ist im ATLS-Konzept Bestandteil des Atemwegsmanagements und kann durch eine später erfolgende Lagerung auf einer Vakuummatratze ergänzt werden.

### B – Breathing

Bei der Überprüfung der **Atmung**

Atmung

müssen **thorakale Spannungszustände**

thorakale Spannungszustände

detektiert werden. Typische klinische Zeichen sind ein fehlendes Atemgeräusch, schwere respiratorische bzw. zirkulatorische Störungen, die durch die Einlage einer **Thoraxdrainage**

Thoraxdrainage

oder ggf. einer **Minithorakotomie**

Minithorakotomie

entlastet werden müssen.

### C – Circulation

Protrahierter Blutverlust und hämorrhagischer Schock sind wichtige Faktoren für die Entstehung der akuten **traumainduzierten Koagulopathie**

traumainduzierten Koagulopathie

und häufige Ursachen des akuten C‑Problems. Bei Traumapatienten wird ein **Monitoring**

Monitoring

(HF, Blutdruck, AF, S_p_O_2_, ggf. endtidales Kohlendioxid [etCO_2_]) etabliert und auf Zeichen einer **Zentralisation**

Zentralisation

geachtet. Nach der Anlage venöser Zugänge erfolgt die Einleitung einer bedarfsgerechten **Volumentherapie**

Volumentherapie,

ggf. ergänzt durch **Vasopressoren**

Vasopressoren

zur Kreislaufunterstützung. Ist es nicht möglich, einen venösen Zugang zu etablieren, ist die Anlage eines intraossären Zugangs empfohlen [8]. Thorax und Abdomen werden untersucht sowie auf äußere und innere Blutverluste geachtet. Es erfolgt eine **Stabilitätsprüfung**

Stabilitätsprüfung

des Beckens und der Femora. Zeigt sich das Becken instabil, muss ein **Beckengurt**

**Figure 2:**
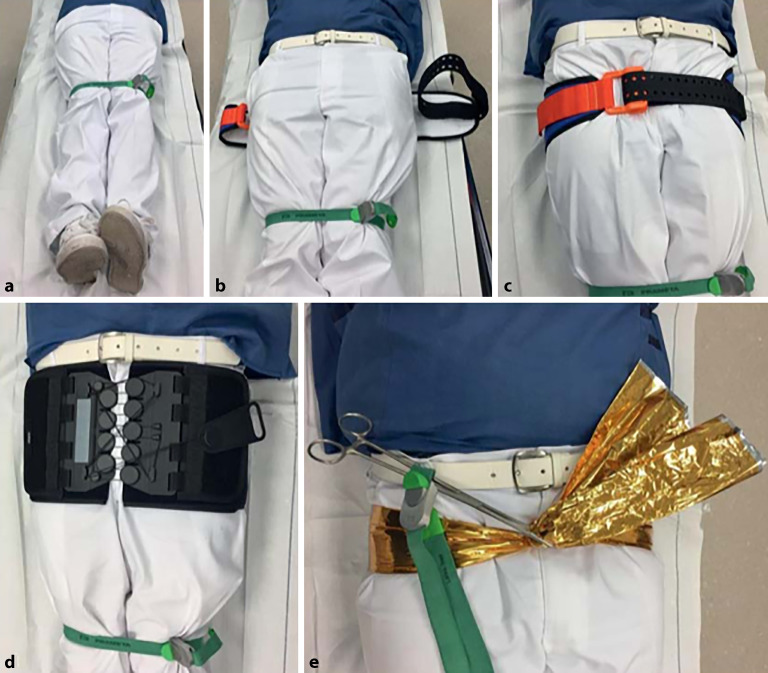

Beckengurt

angelegt werden (Abb. 2).

#### Cave

Unabhängig von der Art des verwendeten Beckengurts sollte seine Mitte über den Trochanteren zu liegen kommen.

#### Merke

- Ziel ist eine annähernd anatomische Reposition.
- Eine zu starke Überkompression sollte vermieden werden.

**Frakturen**

Frakturen

der Extremitäten sollten klinisch achsengerade reponiert und mithilfe einer **Schienenanlage**

Schienenanlage

ruhiggestellt werden, um einen weiteren Blutverlust zu minimieren. Bei unkontrollierbaren intrathorakalen oder intraabdominellen Blutung sollte eine innerklinische chirurgische Therapie so rasch wie möglich erfolgen und nicht durch prähospitale Maßnahmen verzögert werden. Eine moderate Volumentherapie mit einer **„kontrollierten Hypotension“**

„kontrollierten Hypotension“

und einem systolischen Blutdruckwert um 90 mm Hg bei Patienten ohne SHT sollte angestrebt werden. Sofern präklinisch verfügbar, ist eine orientierende **sonographische Untersuchung**

sonographische Untersuchung

im Sinne eines Extended Focused Assessment with Sonography in Trauma (eFAST) möglich bzw. empfohlen, da ein ausreichend erfahrener Anwender wertvolle Informationen bei kreislaufinstabilen Patienten gewinnen kann (z. B. Erkennen von Pneumothorax oder Perikardtamponade).

### D – Disability

Die **neurologische Beurteilung**

neurologische Beurteilung

des Patienten (D – Disability) umfasst die Bewertung der **Vigilanz**

Vigilanz

mit Erhebung der Glasgow Coma Scale (GCS), des Pupillenstatus sowie von Motorik/Sensibilität der Extremitäten. Diese durch das Rettungsteam erhobenen Befunde haben erhebliche Wichtung in der Abwägung der ersten operativen Phase der innerklinischen Weiterversorgung.

### E – Exposure

Im Anschluss erfolgen eine kurze **körperliche Untersuchung**

körperliche Untersuchung,

einschließlich der Körperrückseite, und die Erfassung der **Begleitumstände**

Begleitumstände

(E – Exposure). Im Fokus stehen **Wärmeerhalt**

Wärmeerhalt

und die Vermeidung einer Hypothermie als ein bedeutender Faktor der traumainduzierten Koagulopathie.

Mithilfe dieses Konzepts ist es dem Rettungsteam möglich, in zeitkritischer Einsatzsituation vitale Bedrohungen zu erkennen, prioritätenorientiert einen schwer bzw. schwerstverletzten Patienten zu behandeln und ein Überleben zu ermöglichen.

## Präklinisches Blutungsmanagement

Das präklinische Blutungsmanagement beim Polytrauma umfasst zwei Säulen: die systemischen medikamentösen sowie die lokalen mechanischen Optionen mit Ergänzung lokal wirksamer **Hämostyptika**

Hämostyptika.

Patienten mit lebensbedrohlichen Blutungen soll 1 g **Tranexamsäure**

Tranexamsäure

i.v., idealerweise über 10 min, verabreicht werden [1, 16, 17, 18]. An wenigen Rettungsdienststandorten in Deutschland besteht zudem die Möglichkeit, präklinisch **Erythrozytenkonzentrate**

Erythrozytenkonzentrate

der Blutgruppe 0 oder lyophilisiertes Plasma zu verabreichen. Bei besonders kritisch verletzten Personen kann dies einen Überlebensvorteil bieten; die Verfügbarkeit sollte jedoch speziell geschulten Einsatzteams vorbehalten bleiben.

Große Wunden, v. a. bei Beteiligung der thorakalen oder abdominellen Körperhöhlen sollen mit lokal anwendbaren Hämostyptika versorgt und, sofern möglich, mithilfe eines **Druckverbands**

Druckverbands

temporär verschlossen werden.

Blutende Extremitätenverletzungen sollen gemäß S3-Leitlinie stufenartig folgendermaßen versorgt werden: 1. Druckverband, 2. Kompressionsverband (wenn möglich in Kombination mit einem Hämostyptikum), 3. Tourniquet [1, 10, 19]. Die suffiziente Anlage eines Tourniquets bewirkt eine vollständige Ischämie der distal gelegenen Extremität und muss gut abgewogen werden. Lassen sich große Wunden mithilfe eines Druckverbands versorgen, wird der Blutverlust gestoppt, gleichzeitig die Extremität jedoch weiter durchblutet. Zudem muss bedacht werden, dass bei anliegendem Tourniquet starke Schmerzen (Ischämieschmerz) entsprechend analgetisch adressiert werden müssen.

### Merke

- Nicht jede blutende Extremitätenwunde muss mit einem Tourniquet versorgt werden.
- Ein suffizienter Druckverband ist häufig ausreichend und erleichtert das innerklinische Zeitmanagement.

Wie bereits vorab erwähnt, erreicht die Anlage eines Beckengurts bei V. a. instabile Beckenverletzungen ein signifikant besseres Überleben der polytraumatisierten Patienten [20]. Die Reposition und Schienenanlage von Frakturen großer Röhrenknochen verringern zusätzlich den Blutverlust.

## Rettungsmittel und Zielklinik

Sofern durch die Leitstelle nicht bereits alarmiert, ist die (Nach‑)Alarmierung unterstützender **Luftrettung**

Luftrettung

frühzeitig zu überlegen [1]. Die Studienlage ist nicht eindeutig, es konnte jedoch in einigen Studien ein signifikanter Überlebensvorteil polytraumatisierter Patienten nach Einsatz der Luftrettung nachgewiesen werden [21, 22]. Sollten weitere Transportwege zurückzulegen sein, ergibt sich durch den luftgebundenen Transport zudem ein Zeitvorteil gegenüber der **Bodenrettung**

Bodenrettung.

Polytraumatisierte Patienten sollten nach Möglichkeit in ein regionales oder überregionales **Traumazentrum**

Traumazentrum

transportiert werden [1]. Ist der Patient am Unfallort kardiopulmonal hoch instabil, sollte zunächst der Transport zum nächstmöglichen Krankenhaus in Erwägung gezogen werden. Die Infoboxen 1 und 2 fassen die aktuellen Indikationen zur Alarmierung eines Schockraumteams in der jeweiligen Zielklinik zusammen.

### Infobox 1 Empfehlung zur Schockraumalarmierung, Empfehlungsgrad A^a^

Bei folgenden pathologischen Befunden nach Trauma soll das Schockraumteam aktiviert werden:

A-/B-Problem

- Atemstörungen (S_p_O_2_ < 90 %)/erforderliche Atemwegssicherung
- Atemfrequenz < 10 Atemzüge/min oder > 29 Atemzüge/min

C‑Problem

- Systolischer Blutdruckwert < 90 mm Hg
- Herzfrequenz > 120 Schläge/min
- Schockindex > 0,9
- Positiver Befund im Extended Focused Assessment with Sonography in Trauma (eFAST)

D‑Problem

- Glasgow Coma Scale (GCS) ≤ 12 Punkte

E‑Problem

- Hypothermie < 35,0 °C

Bei folgenden Verletzungen oder Maßnahmen nach Trauma soll das Schockraumteam aktiviert werden:

- Instabiler Thorax
- Mechanisch instabile Beckenverletzung
- Vorliegen von penetrierenden Verletzungen der Rumpf-Hals-Region
- Amputationsverletzung proximal der Hände/Füße
- Sensomotorisches Defizit nach Wirbelsäulenverletzung
- Prähospitale Intervention (erforderliche Atemwegssicherung, Thoraxentlastung, Katecholamingabe, Perikardiozentese, Anlage eines Tourniquets)

^a^Aus Deutsche Gesellschaft für Unfallchirurgie e. V. [1]

### Infobox 2 Empfehlung zur Schockraumalarmierung, Empfehlungsgrad B^a^

Bei folgenden Verletzungen nach Trauma sollte das Schockraumteam aktiviert werden:

- Frakturen von 2 oder mehr proximalen großen Röhrenknochen
- Verbrennungen > 20% und Grad ≥ 2b

Bei folgenden zusätzlichen Kriterien sollte das Trauma‑/Schockraumteam aktiviert werden:

- (Ab)Sturz aus über 3 m Höhe
- Verkehrsunfall (VU) mit Ejektion aus dem Fahrzeug oder Fraktur langer Röhrenknochen

Die Schockraumalarmierung bei geriatrischen Patienten nach relevantem Trauma sollte zusätzlich bei einem der folgenden Parameter erfolgen:

- Systolischer Blutdruckwert < 100 mm Hg
- Bekanntes oder vermutetes Schädel-Hirn-Trauma und Glasgow Coma Scale (GCS) ≤ 14 Punkte
- Zwei oder mehr verletzte Körperregionen
- Fraktur eines oder mehrerer langer Röhrenknochen nach Verkehrsunfall

^a^Aus Deutsche Gesellschaft für Unfallchirurgie e. V. [1]

## Traumareanimation

Die traumabedingte Reanimation unterliegt grundsätzlich dem gleichen cABCDE-Schema wie die Versorgung eines kardiopulmonal stabilen Polytraumas. Nach Etablierung einer Atemwegssicherung müssen thorakale Spannungszustände beseitigt werden. Die Anlage beidseitiger Thoraxdrainagen ist dringend empfohlen [23]. Laut aktualisierter Leitlinie wird empfohlen, diese Maßnahme vor dem Beginn der Thoraxkompression durchzuführen [1]. Bei Verdacht auf eine Perikardtamponade sollte entsprechend den Empfehlungen nach ATLS, des European Resuscitation Council sowie der S3-Leitlinie Polytrauma/Schwerverletzten-Behandlung die Entlastung des Perikards mithilfe einer **Notfallthorakotomie**

**Figure 3:**
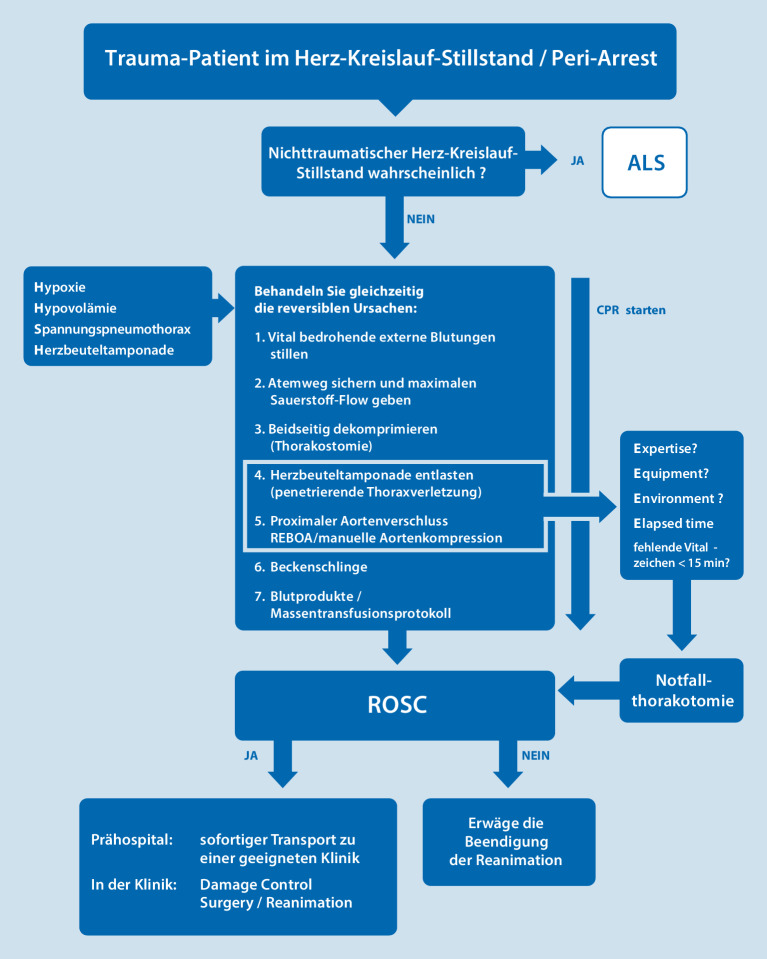

Notfallthorakotomie

erfolgen, weil die Punktion des Perikards häufig u. a. wegen ausgeprägter Blutkoagel keine suffiziente Entlastung zulässt. Nur wenn eine Thorakotomie nicht möglich ist, kann eine möglichst **ultraschallgesteuerte Punktion**

ultraschallgesteuerte Punktion

erwogen werden [24]. Ist es trotz Durchführung dieser Maßnahmen nicht möglich, ein „return of spontaneous circulation“ (ROSC) zu generieren, kann über einen Therapieabbruch nachgedacht werden [1]. Ein Behandlungsalgorithmus zur präklinischen Traumareanimation ist in Abb. 3 dargestellt. In Zweifelsfällen ist der Transport unter Reanimation in die nächstmögliche Klinik zu empfehlen.

Die Durchführung einer Clamshell-Thorakotomie (quer verlaufender Schnitt zur notfallmäßigen chirurgischen Eröffnung aller thorakalen Höhlen auf Höhe des Xiphoids) oder die Anwendung des Systems „resuscitative endovascular balloon occlusion oft he aorta“ (REBOA) ist nicht standardmäßig an der Unfallstelle zu empfehlen und sollte speziell geschultem Personal vorbehalten sein [1].

### Merke

Bei Traumareanimationen gilt:

- externe Blutung stillen,
- Oxygenierung sichern,
- Thorax beidseits dekomprimieren,
- ggf. Perikardtamponade entlasten,
- Becken stabilisieren.

## Fazit für die Praxis

- Die präklinische Versorgung eines Polytraumapatienten ist kein Routineeinsatz; die Behandlungsschritte und Notfallinterventionen müssen regelhaft trainiert werden.
- Das Schema Advanced Trauma Life Support (ATLS) gibt eine strukturierte Handlungsempfehlung zu prä- und innerklinischer Erstversorgung schwer und schwerstverletzter Patienten und muss allen Teammitgliedern bekannt sein.
- Das Stillen von aktiven Blutungen hat hohe Priorität. Blutungen müssen schnell erkannt und direkt oder indirekt adressiert werden. Häufig bedarf es einer Kombination aus systemischer und lokaler Applikation von Hämostyptika sowie der Anlage von suffizienten Kompressionsverbänden, Tourniquets und eines Beckengurts.
- Bereits frühzeitig sind die Zielklinik mit Alarmierung eines Schockraumteams und das entsprechendes Rettungsmittel auszuwählen, um den Patienten bestmöglich zu versorgen und sicher in eine geeignete Zielklinik zu transportieren.

## CME-Fragebogen

In der aktualisierten S3-Leitlinie zur Polytraumaversorgung werden Vorgaben zur Versorgung polytraumatisierter Patienten gegeben. Welche Versorgungsstrategie entspricht der Leitlinienvorgabe?

Jede sichtbare Blutung sollte sofort versorgt werden.

Die Luftrettung sollte nur bei Wirbelsäulenverletzungen aktiviert werden.

Das Behandlungskonzept richtet sich individualisiert nach der Schwere der jeweiligen Einzelverletzungen.

Eine Kapnometrie sollte zur Verifizierung der Atemwegssicherheit standardisiert eingesetzt werden.

Zur Anlage eines Beckengurts gilt die Spina iliaca anterior superior als anatomische Landmarke.

Bei der Versorgung eines polytraumatisierten Patienten zeigt sich eine Hypoxie von 70 %. Wie lässt sich dieses am ehesten erklären?

Einseitiger Spannungspneumothorax

Hintere Beckenringfraktur

14 Punkte auf der Glasgow Coma Scale (GCS) 

Instabile Fraktur des 3. Lendenwirbelkörpers (LWK 3)

Contusio cordis

Nach welcher Reihenfolge sollten die genannten Verletzungen im Rahmen einer präklinischen Erstversorgung behandelt werden?

Instabiles Becken – kritische Blutung aus Amputationswunde am Unterschenkel – zunehmende Atemwegsverlegung – Pneumothorax

Kritische Blutung aus Amputationswunde am Unterschenkel – zunehmende Atemwegsverlegung – Pneumothorax – instabiles Becken

Zunehmende Atemwegsverlegung – Pneumothorax – instabiles Becken – kritische Blutung aus Amputationswunde am Unterschenkel

Pneumothorax – instabiles Becken – kritische Blutung aus Amputationswunde am Unterschenkel – zunehmende Atemwegsverlegung

Instabiles Becken – kritische Blutung aus Amputationswunde am Unterschenkel – Pneumothorax – zunehmende Atemwegsverlegung

Wie sollte die „Traumareanimation“ durchgeführt werden?

Nach der Thoraxkompression wird eine thorakale Entlastungspunktion durchgeführt.

Bei einem A‑Problem sollte eine Clamshell-Thorakotomie erfolgen.

Eine Traumareanimation ohne Generierung eines „return of spontaneous circulation“ (ROSC) kann nach dem Ausschluss reversibler Ursachen präklinisch eingestellt werden.

Reversible Ursachen der Traumareanimation können rein medikamentös behandelt werden.

Bei einem C‑Problem erfolgt standardmäßig die Anwendung der „resuscitative endovascular balloon occlusion of the aorta“ (REBOA).

Bei der Versorgung eines polytraumatisierten Patienten zeigen sich ein gespanntes Abdomen, ein instabiles Becken sowie eine schwere Weichteilverletzungen im Bereich der unteren Extremitäten. Welche Maßnahme beinhaltet das präklinische Blutungsmanagement?

Die lokale Anwendung von Tranexamsäure

Die Gabe von Prothrombinkomplexkonzentrat (Prothrombin, Prokonvertin, Stuart-Prower-Faktor, antihämophiles Globulin B [PPSB])

Die Infusion von Kalzium und Kalium

Die Anlage eines Kompressionsverbandes

Die sofortige Anlage eines Tourniquets

Welches wäre das priorisierte Krankenhaus für einen polytraumatisierten, aber kardiopulmonalen stabilen Patienten (gleiche Verfügbarkeit von boden- und luftgebundenem Transport)?

Lokales Traumazentrum mit 5 min bodengebundener Transportzeit

Überregionales Traumazentrum mit 30 min bodengebundener Transportzeit

Regionales Traumazentrum mit 20 min bodengebundener Transportzeit

Überregionales Traumazentrum mit 10 min luftgebundener Transportzeit

Lokales Traumazentrum mit 10 min luftgebundener Transportzeit

Das Konzept des Advanced Trauma Life Support (ATLS) stellt den Goldstandard in der prä- und innerklinischen Versorgung polytraumatisierter Patienten dar. In welcher Behandlungsreihenfolge wird dieses korrekt durchgeführt?

Der erste Schritt ist die Überprüfung der Beckenstabilität.

Das Atemwegsmanagement erfolgt nach der Reposition von Frakturen der Extremitäten.

Der Patient wird sofort komplett entkleidet, um alle Verletzungen zu erfassen.

Die Untersuchung der Körperrückseite wird vor der neurologischen Beurteilung durchgeführt.

Das Konzept vermittelt die Maßgabe: „Treat first what kills first.“

Welche der folgenden Paarungen aus Verletzung und Versorgungsstrategie ist korrekt?

Instabile Beckenringfraktur – alleinige Gabe von Tranexamsäure

Kritische Blutung nach traumatischer Amputation des Oberschenkels – Anlage eines Tourniquets

Hypoxie und einseitiges fehlendes Atemgeräusch – beidseitige Anlage einer Thoraxdrainage

Starke venöse Blutung aus handtellergroßer Wunde am Oberschenkel – Umstechung und Ligatur am Unfallort

Offene Femur- und Tibiafraktur – Anlage eines Fixateur externe durch den Notarzt

Welche Untersuchung erfolgt unter dem Punkt D (Disability) nach dem Konzept des Advanced Trauma Life Support (ATLS)?

Prüfung der Nackensteifigkeit bei unklarer Vigilanzminderung

Prüfung der Sensibilität und Motorik der oberen und unteren Extremität

Prüfung des Patellarsehnenreflexes

Prüfung der Spitz-Stumpf-Diskrimination

Prüfung des Gleichgewichtssinnes im Romberg-Stehversuch

Wodurch kann die Mortalität eines polytraumatisierten Patienten positiv beeinflusst werden?

Durch präklinische Versorgung aller sichtbaren Verletzungen eines Patienten

Durch regelmäßig und intensiv geschulte Teams in der Anwendung präklinischer Notfallalgorithmen und -eingriffe

Durch bewusst herbeigeführte Hypothermie zur Neuroprotektion beim schweren Schädel-Hirn-Trauma (SHT)

Durch Vermeidung notwendiger präklinische Notfalleingriffe, um keinen „second hit“ zu erzeugen

Durch schonenden bodengebundenen Transport des polytraumatisierten Patienten auch über weite Strecken hinweg
